# 1305. Antimicrobial Activity of Ceftaroline and Comparator Agents against Ceftriaxone-Non-Susceptible *Streptococcus pneumoniae* from the United States (2008-2020)

**DOI:** 10.1093/ofid/ofab466.1497

**Published:** 2021-12-04

**Authors:** Helio S Sader, Mariana Castanheira, Mariana Castanheira, Cecilia G Carvalhaes, S J Ryan Arends, Rodrigo E Mendes

**Affiliations:** 1 JMI Laboratories, North Liberty, Iowa; 2 JMI Laboratories, Inc., North Liberty, Iowa

## Abstract

**Background:**

Ceftaroline (CPT) is a broad-spectrum cephalosporin with activity against *S. pneumoniae* (SPN), including multidrug-resistant (MDR) strains. CPT fosamil is approved for clinical use in the United States (US) to treat community-acquired bacterial pneumonia (CABP). We evaluated the activity of CPT against clinical isolates of ceftriaxone-non-susceptible (CRO-NS) SPN from US medical centers.

**Methods:**

21,750 SPN isolates were consecutively collected (1 per patient) from 201 medical centers in 2008-2020 and tested for susceptibility (S) to CPT and comparator agents using CLSI broth microdilution methods. Among these isolates, 1,419 (6.5%) were CRO-NS (CRO MIC ≥2 mg/L). Other resistant subgroups analyzed included MDR (NS to ≥3 classes of agents; n=4,454) and extensively drug resistant (XDR; NS to ≥5 classes; n=1,708) isolates.

**Results:**

CRO susceptibility varied from 86.9% in 2009 to 98.8% in 2019 and increased from 89.0% in the 2008-2011 period to 98.1% in the 2018-2020 period. CPT was active against 99.9% of CRO-NS SPN (MIC_50/90_, 0.25/0.25 mg/L); only 1 isolate had a CPT MIC ≥0.5 mg/L. The CPT-NS isolate exhibited CPT and CRO MIC values of 1 and 8 mg/L, respectively, and had multiple substitutions in the penicillin binding proteins (PBP), mainly PBP2x, when compared with reference sequences. Also, it showed 31 amino acid alterations in MurM. The most active comparator agents against CRO-NS SPN were linezolid (MIC_50/90_, 0.5/1 mg/L; 100.0%S), levofloxacin (MIC_50/90_, 1/1 mg/L; 98.1%S), tigecycline (MIC_50/90_, ≤0.03/0.06 mg/L; 95.5%S), and vancomycin (MIC_50/90_, ≤1/≤1 mg/L; 100.0%S). Overall, 20.5% of isolates were MDR and 7.9% were XDR. MDR and XDR rates decreased from 24.4% and 13.5% in 2008-2011 to 16.8% and 2.4% in 2018-2020, respectively. CPT retained potent activity against MDR (MIC_50/90_, 0.12/0.25 mg/L; 99.9%S) and XDR (MIC_50/90_, 0.25/0.25 mg/L; 100.0%S) isolates; whereas CRO exhibited limited activity against both MDR (MIC_50/90_, 1/2 mg/L; 68.9%S) and XDR (MIC_50/90_, 2/2 mg/L; 26.7%S) isolate subsets. Among CRO-NS isolates, 97.7% were MDR and 88.2% were XDR.

**Conclusion:**

CPT demonstrated potent and consistent activity over time (2008-2020) against a large collection of SPN from US medical centers, including CRO-NS, MDR, and XDR isolates.

**Disclosures:**

**Helio S. Sader, MD, PhD, FIDSA**, **AbbVie (formerly Allergan**) (Research Grant or Support)**Basilea Pharmaceutica International, Ltd.** (Research Grant or Support)**Cipla Therapeutics** (Research Grant or Support)**Cipla USA Inc.** (Research Grant or Support)**Department of Health and Human Services** (Research Grant or Support, Contract no. HHSO100201600002C)**Melinta Therapeutics, LLC** (Research Grant or Support)**Nabriva Therapeutics** (Research Grant or Support)**Pfizer, Inc.** (Research Grant or Support)**Shionogi** (Research Grant or Support)**Spero Therapeutics** (Research Grant or Support) **Mariana Castanheira, PhD**, **AbbVie (formerly Allergan**) (Research Grant or Support)**Bravos Biosciences** (Research Grant or Support)**Cidara Therapeutics, Inc.** (Research Grant or Support)**Cipla Therapeutics** (Research Grant or Support)**Cipla USA Inc.** (Research Grant or Support)**GlaxoSmithKline** (Research Grant or Support)**Melinta Therapeutics, Inc.** (Research Grant or Support)**Melinta Therapeutics, LLC** (Research Grant or Support)**Pfizer, Inc.** (Research Grant or Support)**Qpex Biopharma** (Research Grant or Support)**Shionogi** (Research Grant or Support)**Spero Therapeutics** (Research Grant or Support) **Mariana Castanheira, PhD**, Affinity Biosensors (Individual(s) Involved: Self): Research Grant or Support; Allergan (Individual(s) Involved: Self): Research Grant or Support; Amicrobe, Inc (Individual(s) Involved: Self): Research Grant or Support; Amplyx Pharma (Individual(s) Involved: Self): Research Grant or Support; Artugen Therapeutics USA, Inc. (Individual(s) Involved: Self): Research Grant or Support; Astellas (Individual(s) Involved: Self): Research Grant or Support; Basilea (Individual(s) Involved: Self): Research Grant or Support; Beth Israel Deaconess Medical Center (Individual(s) Involved: Self): Research Grant or Support; BIDMC (Individual(s) Involved: Self): Research Grant or Support; bioMerieux Inc. (Individual(s) Involved: Self): Research Grant or Support; BioVersys Ag (Individual(s) Involved: Self): Research Grant or Support; Bugworks (Individual(s) Involved: Self): Research Grant or Support; Cidara (Individual(s) Involved: Self): Research Grant or Support; Cipla (Individual(s) Involved: Self): Research Grant or Support; Contrafect (Individual(s) Involved: Self): Research Grant or Support; Cormedix (Individual(s) Involved: Self): Research Grant or Support; Crestone, Inc. (Individual(s) Involved: Self): Research Grant or Support; Curza (Individual(s) Involved: Self): Research Grant or Support; CXC7 (Individual(s) Involved: Self): Research Grant or Support; Entasis (Individual(s) Involved: Self): Research Grant or Support; Fedora Pharmaceutical (Individual(s) Involved: Self): Research Grant or Support; Fimbrion Therapeutics (Individual(s) Involved: Self): Research Grant or Support; Fox Chase (Individual(s) Involved: Self): Research Grant or Support; GlaxoSmithKline (Individual(s) Involved: Self): Research Grant or Support; Guardian Therapeutics (Individual(s) Involved: Self): Research Grant or Support; Hardy Diagnostics (Individual(s) Involved: Self): Research Grant or Support; IHMA (Individual(s) Involved: Self): Research Grant or Support; Janssen Research & Development (Individual(s) Involved: Self): Research Grant or Support; Johnson & Johnson (Individual(s) Involved: Self): Research Grant or Support; Kaleido Biosceinces (Individual(s) Involved: Self): Research Grant or Support; KBP Biosciences (Individual(s) Involved: Self): Research Grant or Support; Luminex (Individual(s) Involved: Self): Research Grant or Support; Matrivax (Individual(s) Involved: Self): Research Grant or Support; Mayo Clinic (Individual(s) Involved: Self): Research Grant or Support; Medpace (Individual(s) Involved: Self): Research Grant or Support; Meiji Seika Pharma Co., Ltd. (Individual(s) Involved: Self): Research Grant or Support; Melinta (Individual(s) Involved: Self): Research Grant or Support; Menarini (Individual(s) Involved: Self): Research Grant or Support; Merck (Individual(s) Involved: Self): Research Grant or Support; Meridian Bioscience Inc. (Individual(s) Involved: Self): Research Grant or Support; Micromyx (Individual(s) Involved: Self): Research Grant or Support; MicuRx (Individual(s) Involved: Self): Research Grant or Support; N8 Medical (Individual(s) Involved: Self): Research Grant or Support; Nabriva (Individual(s) Involved: Self): Research Grant or Support; National Institutes of Health (Individual(s) Involved: Self): Research Grant or Support; National University of Singapore (Individual(s) Involved: Self): Research Grant or Support; North Bristol NHS Trust (Individual(s) Involved: Self): Research Grant or Support; Novome Biotechnologies (Individual(s) Involved: Self): Research Grant or Support; Paratek (Individual(s) Involved: Self): Research Grant or Support; Pfizer (Individual(s) Involved: Self): Research Grant or Support; Prokaryotics Inc. (Individual(s) Involved: Self): Research Grant or Support; QPEX Biopharma (Individual(s) Involved: Self): Research Grant or Support; Rhode Island Hospital (Individual(s) Involved: Self): Research Grant or Support; RIHML (Individual(s) Involved: Self): Research Grant or Support; Roche (Individual(s) Involved: Self): Research Grant or Support; Roivant (Individual(s) Involved: Self): Research Grant or Support; Salvat (Individual(s) Involved: Self): Research Grant or Support; Scynexis (Individual(s) Involved: Self): Research Grant or Support; SeLux Diagnostics (Individual(s) Involved: Self): Research Grant or Support; Shionogi (Individual(s) Involved: Self): Research Grant or Support; Specific Diagnostics (Individual(s) Involved: Self): Research Grant or Support; Spero (Individual(s) Involved: Self): Research Grant or Support; SuperTrans Medical LT (Individual(s) Involved: Self): Research Grant or Support; T2 Biosystems (Individual(s) Involved: Self): Research Grant or Support; The University of Queensland (Individual(s) Involved: Self): Research Grant or Support; Thermo Fisher Scientific (Individual(s) Involved: Self): Research Grant or Support; Tufts Medical Center (Individual(s) Involved: Self): Research Grant or Support; Universite de Sherbrooke (Individual(s) Involved: Self): Research Grant or Support; University of Iowa (Individual(s) Involved: Self): Research Grant or Support; University of Iowa Hospitals and Clinics (Individual(s) Involved: Self): Research Grant or Support; University of Wisconsin (Individual(s) Involved: Self): Research Grant or Support; UNT System College of Pharmacy (Individual(s) Involved: Self): Research Grant or Support; URMC (Individual(s) Involved: Self): Research Grant or Support; UT Southwestern (Individual(s) Involved: Self): Research Grant or Support; VenatoRx (Individual(s) Involved: Self): Research Grant or Support; Viosera Therapeutics (Individual(s) Involved: Self): Research Grant or Support; Wayne State University (Individual(s) Involved: Self): Research Grant or Support **Cecilia G. Carvalhaes, MD, PhD**, **AbbVie (formerly Allergan**) (Research Grant or Support)**Cidara Therapeutics, Inc.** (Research Grant or Support)**Cipla Therapeutics** (Research Grant or Support)**Cipla USA Inc.** (Research Grant or Support)**Melinta Therapeutics, LLC** (Research Grant or Support)**Pfizer, Inc.** (Research Grant or Support) **S J Ryan Arends, PhD**, **AbbVie (formerly Allergan**) (Research Grant or Support)**GlaxoSmithKline, LLC** (Research Grant or Support)**Melinta Therapeutics, LLC** (Research Grant or Support)**Nabriva Therapeutics** (Research Grant or Support)**Spero Therapeutics** (Research Grant or Support) **Rodrigo E. Mendes, PhD**, **AbbVie** (Research Grant or Support)**AbbVie (formerly Allergan**) (Research Grant or Support)**Cipla Therapeutics** (Research Grant or Support)**Cipla USA Inc.** (Research Grant or Support)**ContraFect Corporation** (Research Grant or Support)**GlaxoSmithKline, LLC** (Research Grant or Support)**Melinta Therapeutics, Inc.** (Research Grant or Support)**Melinta Therapeutics, LLC** (Research Grant or Support)**Nabriva Therapeutics** (Research Grant or Support)**Pfizer, Inc.** (Research Grant or Support)**Shionogi** (Research Grant or Support)**Spero Therapeutics** (Research Grant or Support)

